# Concurrent Intracranial and Spinal Subdural Hematoma in a Teenage Athlete: A Case Report of This Rare Entity

**DOI:** 10.1155/2014/143408

**Published:** 2014-09-29

**Authors:** Daniel S. Treister, Sara E. Kingston, Gabriel Zada, Manu Singh, Jesse G. A. Jones, Jena N. Mills, Alexander Lerner, Orest B. Boyko, Meng Law, Anandh Rajamohan, Mark S. Shiroishi

**Affiliations:** ^1^Division of Neuroradiology, Department of Radiology, Keck School of Medicine, University of Southern California, Los Angeles, CA, USA; ^2^Department of Neurological Surgery, Keck School of Medicine, University of Southern California, Los Angeles, CA, USA

## Abstract

A 15-year-old male high school football player presented with episodes of headache and complete body stiffness, especially in the arms, lower back, and thighs, immediately following a football game. This was accompanied by severe nausea and vomiting for several days. Viral meningitis was suspected by the primary clinician, and treatment with corticosteroids was initiated. Over the next several weeks, there was gradual symptom improvement and the patient returned to his baseline clinical status. The patient experienced a severe recurrence of the previous myriad of symptoms following a subsequent football game, without an obvious isolated traumatic episode. In addition, he experienced a new left sided headache, fatigue, and difficulty ambulating. He was admitted and an extensive workup was performed. CT and MRI of the head revealed concurrent intracranial and spinal subdural hematomas (SDH). Clinical workup did not reveal any evidence of coagulopathy or predisposing vascular lesions. Spinal SDH is an uncommon condition whose concurrence with intracranial SDH is an even greater clinical rarity. We suggest that our case represents an acute on chronic intracranial SDH with rebleeding, membrane rupture, and symptomatic redistribution of hematoma to the spinal subdural space.

## 1. Introduction

Spinal subdural hematoma (SDH) is a rare condition with a variety of potential underlying etiologies, which include trauma, coagulopathy, underlying vascular lesions, lumbar puncture, and idiopathic causes [1, 2]. In a recent meta-analysis, SDH represented only 4% of 613 spinal hematoma cases reported between 1826 and 1996, implying that while spinal hematoma has a relatively low incidence, those in the subdural space represent a unique subclassification [2]. The concurrence of intracranial and spinal SDHs is an even greater clinical rarity with only 35 reported cases [3]. We present a case of likely trauma-induced concurrent intracranial and spinal SDHs in a teenage football player.

## 2. Case Report

A 15-year-old high school football player initially presented to his primary care physician complaining of episodic headaches, total body stiffness, and severe nausea and vomiting for several days following participation in a football game. Viral meningitis was suspected, so the patient was started on oral prednisone. Of note, the patient had experienced mild back pain during the week preceding the game and was using ibuprofen for pain management. Symptoms gradually resolved over the next two weeks and the patient returned to baseline clinical status before resuming athletic activities.

Following participation in a subsequent football game, the patient was admitted to the hospital with recurrence and exacerbation of his previous symptoms. At this time, his headache was localized to the left side, body stiffness was most pronounced in the posterior thighs and lower back, and nausea and vomiting were persistent. The patient denied a recent history of fevers and had no evidence of neck stiffness. Neurological exam was unremarkable other than a mildly unsteady gait and did not reveal any focal deficits.

**Figure 1:**
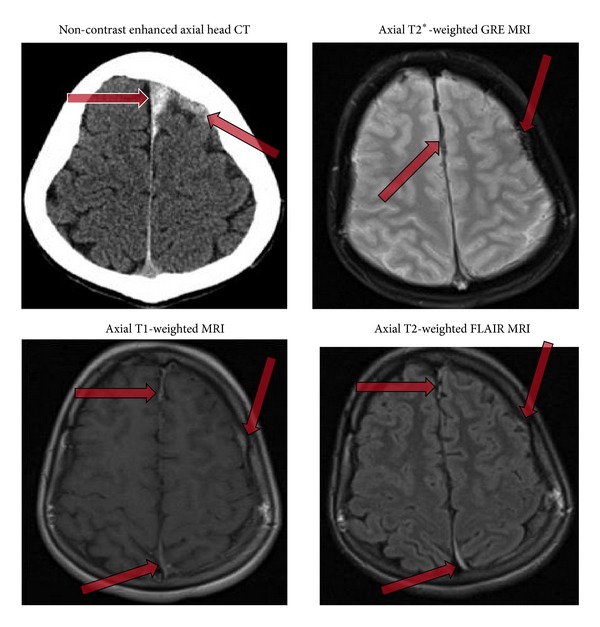
Axial noncontrast CT and MRI of the brain showing evidence of a thin (approximately 2-3 mm) subdural hematoma (arrows) over the left hemisphere with extension into the interhemispheric fissure.

**Figure 2:**
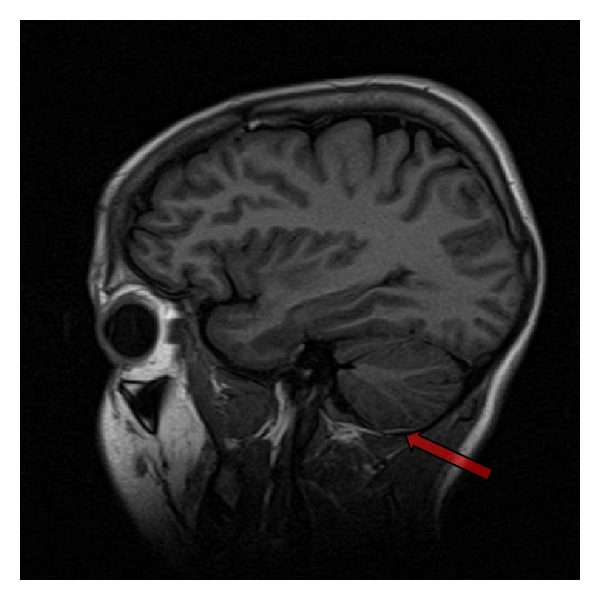
Sagittal T1-weighted image of the brain demonstrates a very thin mildly hyperintense subdural hematoma over the left cerebellar convexity.

**Figure 3:**
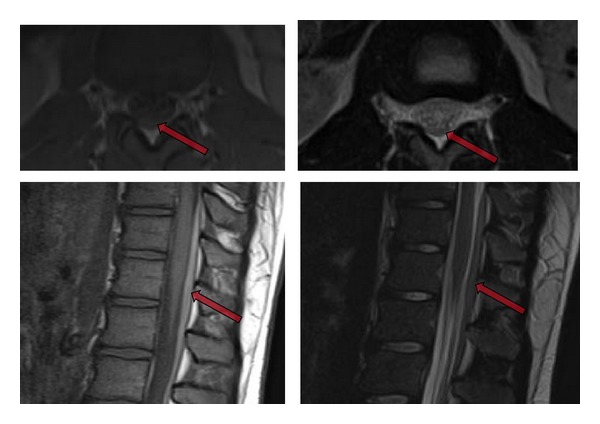
Lumbar spine MRI [Axial and sagittal T1-weighted (left top and bottom); axial and sagittal T2-weighted (right top and bottom) images] demonstrates dorsal subdural hematoma (arrows) extending from approximately T11/12 to L4 levels.

Clinical workup resulted in the diagnosis of intracranial and spinal subdural hematoma. CT and MRI of the head revealed a 2-3 mm thick intracranial subdural hematoma over the left cerebral hemisphere with extension into the interhemispheric fissure (Figure 1). A thin subdural hematoma was also noted over the left cerebellar hemisphere of the posterior fossa (Figure 2). MRI of the lumbar spine demonstrated a 3 mm wide dorsal subdural hematoma extending from L1 to L4 levels without compression of the conus medullaris or cauda equina nerve roots (Figure 3). Laboratory evaluation revealed no evidence of coagulopathy. The patient was managed conservatively and followed up on an outpatient basis following a two-day hospitalization.

Over the next week, the patient's headache and back pain resolved. However, lower limb pain progressed to pain radiating down the posterior thighs and lateral leg and was most pronounced in the right leg. Physical exam at this time was remarkable only for mild weakness of right knee extension and positive right-sided straight leg raise sign. Extensive imaging workup consisting of MRI of cervical and thoracic spine and MR angiography of the brain did not reveal any underlying vascular lesions.

With conservative clinical management, the patient slowly returned to baseline clinical status without residual neurologic deficits. Repeat imaging of the brain and spine 2 months later showed complete resolution of both prior subdural hematomas.

## 3. Discussion

The majority of patients with spinal SDH have a history of trauma or predisposing risk factors including inherited or acquired clotting disorders, iatrogenic spinal instrumentation (e.g., lumbar puncture), or underlying spinal vascular malformation. It has been reported that trauma and hematologic disorders account for 84% of spinal SDH cases [4]. This patient was potentially subject to both trauma and drug-induced coagulopathy. While the patient did not recall a specific traumatic event, his participation in football preceding both instances of symptom onset strongly suggests a contributory role for sports trauma. In addition, the patient was self-medicating with unknown cumulative dose of oral NSAIDs for the week prior to the initial episode of symptoms, so drug-induced platelet dysfunction could have also played a role. Eight hundred milligrams of ibuprofen has antiplatelet effects that last up to 24 hours after ingestion [5]; however, it is unknown if this is related to spinal hematomas. In cases of concurrent intracranial and spinal SDH, a debate exists as to whether the hematomas represent two separate and coincidental events (i.e., both intracranial and spinal sources of subdural bleeding) or are related by migration of blood from the cranium to the spine. While the intracranial subdural space contains bridging veins that are vulnerable to tearing forces, the spinal subdural space lacks an analogous vasculature. This explains, in part, why intracranial SDH has a greater incidence than its spinal counterpart. Low CSF pressure in the spinal subarachnoid space following ventriculoperitoneal (VP) shunt placement or craniotomy has been suggested as a potential mechanism for spinal vessel rupture [3]. With sudden pressure increases in the intrathoracic or intra-abdominal cavities, pressure is transmitted along spinal vessels which are vulnerable to tearing if not neutralized by a compensatory increase in spinal fluid pressure [6]. Additionally, intracranial hypotension from a VP shunt may be a cause of primary intracranial SDH by facilitating widening of the intracranial subdural space, tearing of bridging of veins, and hematoma migration [7, 8]. A review of 35 cases of concurrent intracranial and spinal SDH revealed 6 cases which followed VP shunt placement or craniotomy [3]; however this does not apply to the patient in our case.

A significant number of spinal SDH cases have implicated the intracranial subdural space as the initial site of bleeding with subsequent layering of blood in the dependent areas of the skull and spine, particularly in patients with simultaneous intracranial and spinal subdural SDHs [9–13]. Spinal SDHs with concurrent intracranial SDH are most likely to be found in the most dependent regions of the spine, namely, the lower thoracic and lumbar [14]. Many reports of concurrent intracranial and spinal SDHs include a pattern, as seen in our patient, of initial symptoms implicating intracranial hematoma (headache, nausea, and vomiting) followed by spontaneous resolution of these symptoms and development of spinal symptoms (back pain, radiculopathy, and lower extremity weakness) [12, 15, 16]. Follow-up imaging of patients with acute intracranial SDH has demonstrated a gravity-dependent redistribution of hematoma to the surface of the tentorium and posterior fossa [10–12]. A case report from Lecouvet et al. had an MRI demonstrating continuous hematoma spanning the subdural space from the posterior fossa to the lumbar spine [13]. It is unclear whether chronic intracranial SDH can redistribute in this manner as hematoma mobility is limited by inner and outer membrane formation [1, 11]. However, rebleeding has been suggested as a mechanism for membrane rupture and caudal hematoma redistribution [1]. It is possible that reported cases of symptomatic spinal SDH may only represent a minority of total spinal SDHs with intracranial origins since asymptomatic cases would not be investigated with imaging. However, a recent prospective study of 168 patients with intracranial SDH only revealed 2 patients with concurrent spinal SDH. Both patients in the study had suffered trauma to both the head and hip/lumbar regions, suggesting that so-called “double trauma” is also an important risk factor for concurrent SDH [3]. A major limitation of this prospective study is that it only included patients with chronic intracranial SDH, and the incidence of concurrent spinal SDH may be much greater in cases of acute and subacute intracranial SDH. Our patient had recurrence of intracranial hematoma symptoms with progression of spinal SDH symptoms, radiographic evidence of hematoma extension into the posterior fossa, and lumbar location of spinal SDH. We suggest that our case represents an acute on chronic intracranial SDH with rebleeding, membrane rupture, and symptomatic redistribution of hematoma to the spinal subdural space.

MRI is superior to CT for detecting spinal SDH. Localization of hematoma to the epidural or subdural space can be difficult, but subdural location is confirmed by the presence of dura between the blood products and epidural fat. The differential diagnosis for spinal SDH includes spinal tumor, extramedullary lesions including tumor or abscess, and disc protrusion [15]. The most common location for a spinal SDH is posterolateral, often with mass effect on the nerve roots and cauda equina. When extensive, the mass effect from the SDH may result in the “inverted Mercedes star” appearance with the cauda nerve roots accounting for the lateral projections and the filum terminale the posterior one [13]. Conservative management is appropriate in cases like ours where spinal symptoms are mild and nonprogressive [11]. Surgical spinal decompression may be necessary in more severe cases. It is important to note that in cases of concurrent SDH, the intracranial SDH should be evacuated before the spinal SDH in order to decrease risk of brain herniation [1].
